# Integrated Single Cell Analysis Reveals the Transcriptional Heterogeneity of Mouse Double Negative T Cells

**DOI:** 10.3390/ijms27177966

**Published:** 2026-09-07

**Authors:** Jun Zhao, Jiang Zhu, Jian Zhang

**Affiliations:** School of Medical Informatics, Daqing Campus, Harbin Medical University, Daqing 163319, China; zhaojunbio@163.com (J.Z.);

**Keywords:** double negative T cells, single cell RNA sequencing, immune landscape, single cell data integration, cellular heterogeneity, rare immune cells

## Abstract

Double negative T cells (DNT cells) are a rare T cell population involved in immune regulation, inflammation, autoimmunity, transplantation, and tumor immunity. However, their low abundance and dispersed distribution across tissues have limited a systematic understanding of their cellular organization and transcriptional diversity. To address this problem, we developed an integrative single cell framework to reconstruct the mouse DNT cell landscape across tissues using multiple public single cell RNA sequencing datasets. Using transcriptomic criteria, we identified and integrated 7984 mouse RNA defined DNT cells. The integrated population was resolved into multiple transcriptionally distinct clusters. These clusters were organized into naive like, proinflammatory, cytotoxic, proliferative, and myeloid associated states, revealing that mouse RNA defined DNT cells constitute a highly heterogeneous yet structured transcriptional compartment. Integrated downstream analyses indicated transcriptional relationships among these subsets. Naive like populations showed inferred transcriptional relationships with inflammatory and cytotoxic programs, while virtual knockout and intercellular communication analyses identified distinct predicted regulatory and signaling features across states. To place these mouse DNT associated genes in a human disease context, selected genes were mapped to their corresponding human homologs and examined using TCGA pan cancer transcriptomic data. Together, these findings define a cross tissue transcriptional framework for mouse RNA defined DNT cells and provide a basis for further evaluating the conservation and relevance of these transcriptional programs in human DNT biology. More broadly, this study provides a generalizable integrative strategy for reconstructing and characterizing rare immune cell populations that are insufficiently represented in individual single cell datasets.

## 1. Introduction

Double negative T cells (DNT cells) are a minor population of mature T lymphocytes that lack both CD4 and CD8 expression [1]. Despite their low frequency, they have been reported in peripheral blood, lymphoid organs, and nonlymphoid tissues [2], where they participate in a wide range of immune processes. DNT cells have been associated with immune tolerance, inflammatory regulation, autoimmunity, transplantation, graft versus host disease [3], and antitumor immunity [4]. Their functions vary widely across different immunological and pathological contexts. In some settings, they suppress excessive immune activation or autoreactive responses. In others, they display inflammatory or cytotoxic activity. These contrasting observations support a heterogeneous view of DNT cells comprising multiple functional states [5]. Interest in DNT cells has grown with the advent of high resolution immune profiling. Earlier studies mainly characterized these cells through flow cytometry and functional assays, which established their regulatory and cytotoxic potential but provided limited information about their internal diversity [6]. Single cell RNA sequencing has begun to fill this gap. Recent studies have revealed that DNT cells can express transcriptional programs linked to cytotoxicity, immune activation, regulation, and innate-like responses [7]. These findings have broadened our understanding of the transcriptional diversity within this population. At the same time, they have raised new questions about how these states are organized and whether they represent stable subsets, transient activation states, or tissue adapted phenotypes.

Current knowledge remains incomplete for several reasons. DNT cells are rare, and their representation in individual single cell RNA sequencing datasets is often limited, constraining the detection of rare subsets and comparisons across tissues [8]. This limits the detection of rare subsets and comparisons across tissues. Most published studies have also focused on a specific disease or tissue environment [9]. These studies provide valuable context but do not offer a unified view of the peripheral DNT cell compartment. Consequently, it remains unclear whether the observed variation reflects shared DNT cell biology or local tissue conditions. The relationship among quiescent, inflammatory, proliferative, and effector like states is also poorly understood. A further difficulty lies in the transcriptomic definition of DNT cells. In conventional immunophenotyping, DNT cells are identified by the absence of CD4 and CD8 proteins together with the presence of T cell markers. In single cell RNA sequencing data, however, the absence of Cd4 and Cd8 transcripts can be influenced by low transcript abundance and technical dropout [7]. In addition, recently activated conventional CD4 or CD8 T cells may show reduced or undetected coreceptor transcripts, creating a risk that these cells are included in an RNA based DNT population. Cell identity must therefore be inferred from a combination of positive and negative markers, with coreceptor status evaluated in this broader context. This principle is supported by previous DNT studies. Tian et al. identified human TCRαβ positive DNT cells by combining T cell identity with CD4 and CD8 negativity and additional evaluation of unconventional T cell populations [10]. Yang et al. further assessed experimentally isolated mouse DNT cells using positive CD3 associated transcripts together with negative coreceptor and lineage associated transcripts. Islam et al. identified DN cells within an established mouse T cell population according to CD4, CD8a, and CD8b1 transcript expression, while also examining CD3 expression, transcriptomic feature detection, and Trgv2 expression [8]. A similar T cell anchored strategy was used in a large scale integrated single cell DNT study, in which DNT populations were extracted from an established T cell compartment before downstream analysis [11]. Together, these studies support a general analytical sequence in which T cell identity is established before evaluation of coreceptor status and additional lineage associated signals. However, such RNA based identification does not provide the same certainty as protein level immunophenotyping. This issue becomes especially important when candidate DNT cells express strong inflammatory, cytotoxic, or myeloid associated programs. Such expression patterns may reflect functional adaptation, but they may also complicate annotation. For this reason, transcriptomically selected populations should be interpreted cautiously as RNA defined DNT cells. To obtain a broader view of this rare population, we integrated multiple public mouse single cell RNA sequencing datasets and reconstructed a cross tissue landscape of mouse RNA defined DNT cells. The study was designed to define the major transcriptional states present within this compartment, examine their relationships, and assess whether distinct transcriptional programs could be resolved within a common framework. Because the single cell component of this study was derived from mouse datasets, the identified states primarily represent the transcriptional characteristics of mouse RNA defined DNT cells. To explore whether genes associated with these states may also be relevant in human disease, selected mouse DNT associated genes were mapped to their human homologs and examined separately using TCGA pan cancer transcriptomic data. This comparison was intended to provide a human cancer related context for these genes at the gene expression level. By increasing the number of analyzable cells and combining information across individual datasets, this approach provides a more comprehensive basis for investigating DNT cell heterogeneity. It also offers a general strategy for studying other rare immune cell populations.

## 2. Results

### 2.1. Construction and Quality Assessment of the Mouse RNA Defined DNT Cell Dataset

**Figure 1:**
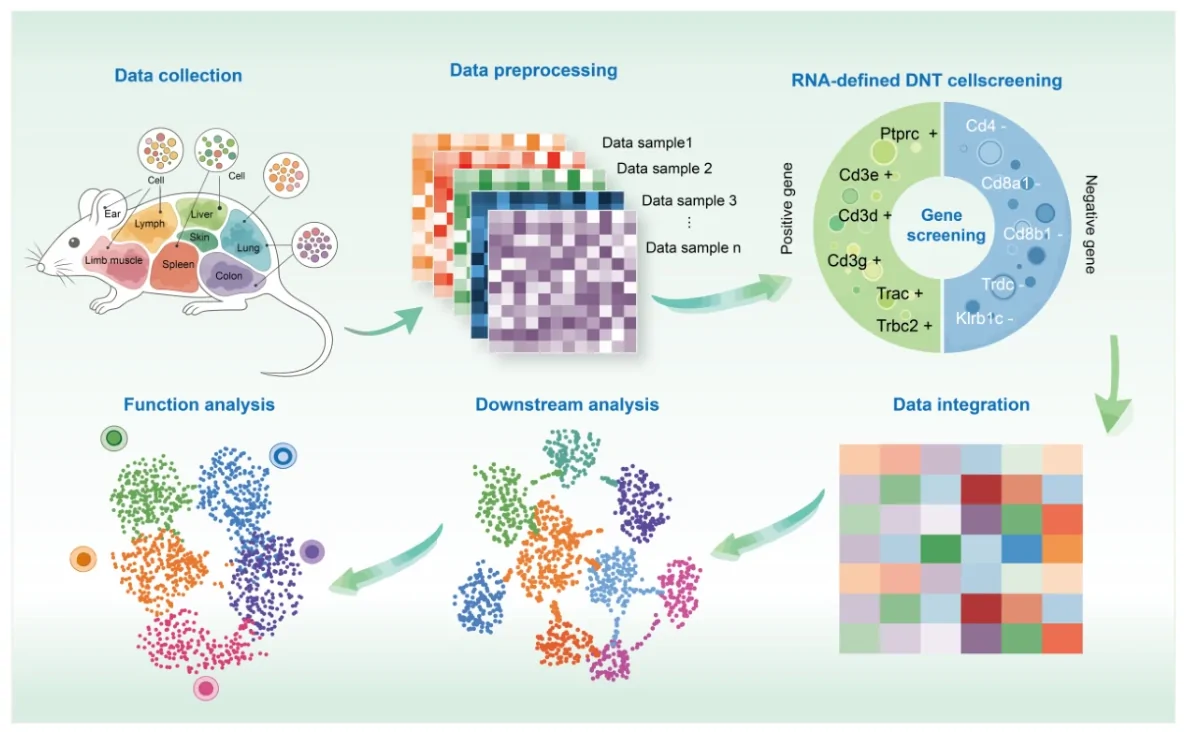
Workflow for construction of the mouse RNA defined DNT cell dataset. The schematic shows data collection, preprocessing, RNA based DNT cell screening using positive and negative markers, data integration, and downstream analysis.

Before integration and downstream analysis, we first constructed and evaluated the mouse RNA defined DNT cell dataset. A total of 66 publicly available mouse GEO samples were included after evaluation of sample information, tissue origin, sequencing platform, expression matrix availability, and associated metadata. These samples represented the colon, ear, limb muscle, liver, lung, lymphoid tissue, skin, and spleen. Because the source datasets differed in cell number, sequencing depth, and transcriptomic complexity, each dataset was processed and evaluated separately before DNT cell screening. Cell quality was assessed using detected gene number, total transcript counts, and mitochondrial transcript proportion. The source datasets are summarized in Supplementary Table S1, and the overall dataset construction and analysis workflow is shown in Figure 1.

Candidate RNA defined DNT cells were then identified separately within each source dataset using the predefined transcriptional criteria. Cells were required to show detectable expression of Ptprc, Cd3d, Cd3e, Cd3g, Trac, and Trbc2, while showing no detectable expression of Cd4, Cd8a, Cd8b1, Trdc, or Klrb1c. Thus, candidate cells were selected using multiple positive T cell associated transcripts together with coreceptor and lineage associated criteria. This sequential assessment followed the same general analytical logic used in previous single cell studies, in which DN or DNT populations were identified within an established T cell compartment before evaluation of additional lineage associated features. The T cell associated transcriptional characteristics of the retained population were further examined using a CD3 module score based on Cd3d, Cd3e, and Cd3g, together with Trac and Trbc2 expression. Potential unconventional T cell associated signals were also evaluated before integration. Among the evaluable transcripts, Trdc, Trgj1, Trgj2, Trgj3, Trgj4, Traj33, and Klrb1c were not detected, whereas Trgv2 and Trav1 were detected only in small subsets of cells. The absence of an individual transcript cannot establish definitive lineage negativity in sparse single cell RNA sequencing data. These analyses were therefore used as transcriptional assessments of the retained population. After removal of Trgv2 positive cells, the major proinflammatory and cytotoxic marker patterns remained detectable within the existing cluster framework, as shown in Supplementary Figure S2A and Supplementary Table S3. After source level quality assessment, RNA based DNT screening, and additional transcriptional evaluation, 7984 mouse RNA defined DNT cells were combined for subsequent integration and biological analysis.

### 2.2. Integrated Clustering and Tissue Distribution of Mouse RNA Defined DNT Cells

**Figure 2:**
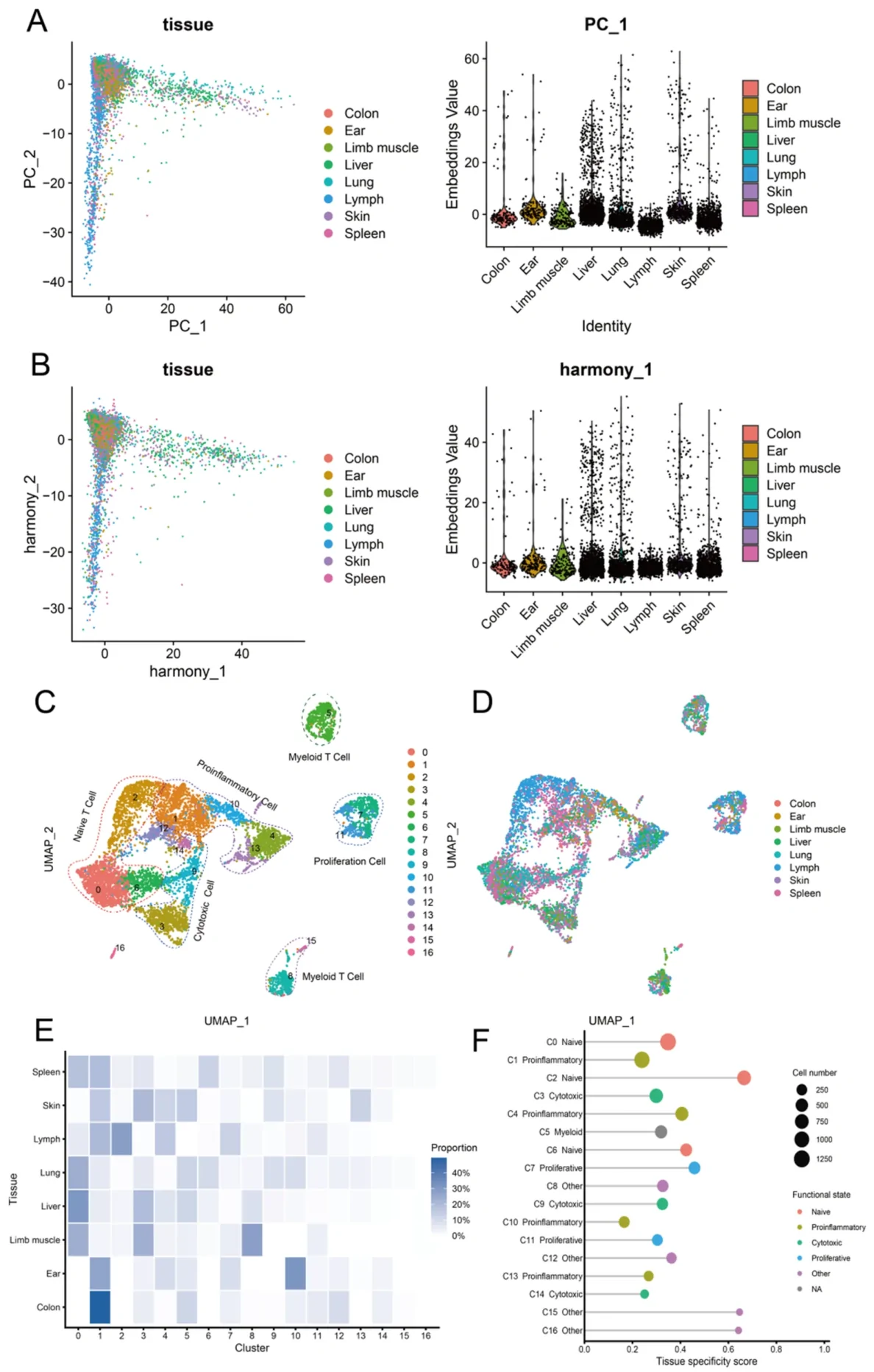
Integrated clustering and tissue distribution of mouse RNA defined DNT cells. (**A**) Principal component representation of DNT cells before Harmony correction, colored by tissue origin. (**B**) Harmony corrected low dimensional representation showing improved integration across tissues. (**C**) UMAP visualization of the 17 transcriptionally distinct DNT cell clusters, with major naive, proinflammatory, cytotoxic, proliferative, and myeloid associated DNT states indicated according to marker based annotation. (**D**) Tissue distribution after integration, showing broad cross tissue overlap together with regional tissue associated enrichment. (**E**) Heatmap showing the within tissue normalized distribution of DNT cells across clusters. (**F**) Tissue specificity scores across DNT clusters, with dot size indicating cluster cell number and color indicating the major transcriptional state. Higher scores indicate a more restricted tissue distribution.

Following construction and quality assessment of the RNA defined DNT population, Harmony based integration was applied to the mouse RNA defined DNT cell dataset, with tissue origin used as the grouping variable to facilitate joint analysis of cells from different tissue sources within a common low dimensional representation, as shown in Figure 2A,B. Following correction, cells from different tissues were distributed within a shared transcriptional space, allowing transcriptionally related cells from different tissue sources to be examined within a common integrated framework. The corrected dataset was subsequently subjected to dimensionality reduction and graph based clustering. In total, 17 transcriptionally distinct mouse DNT cell clusters, cluster 0 to 16, were identified, indicating marked cellular diversity within the mouse DNT compartment, as shown in Figure 2C. Based on the dominant transcriptional programs subsequently identified by marker gene analysis, these clusters were broadly organized into naive, proinflammatory, cytotoxic, proliferative, and myeloid associated DNT states, with several clusters showing overlapping transcriptional features. These state annotations are indicated in Figure 2C to provide an overview of the transcriptional organization of the integrated population. Tissue origin annotation on the integrated UMAP showed that DNT cells from different tissues displayed distinct distribution patterns among the identified clusters, with some clusters containing cells from multiple tissues and others showing apparent tissue associated enrichment, as shown in Figure 2D. The distribution of DNT clusters within each tissue was further examined using within tissue normalized proportions, as shown in Figure 2E. The relative representation of individual clusters differed among tissues, indicating variation in DNT cluster composition across tissue sources. Tissue specificity was then assessed at the cluster level, as shown in Figure 2F. Some clusters showed broader tissue distributions, whereas others were more strongly concentrated in a limited number of tissues. Together, these analyses indicate that the integrated DNT landscape contains transcriptional states with different patterns of representation across tissue sources, including broadly distributed states and states concentrated in a limited number of tissues.

### 2.3. Marker Gene Expression Reveals Distinct Transcriptional States of DNT Cell Subsets

**Figure 3:**
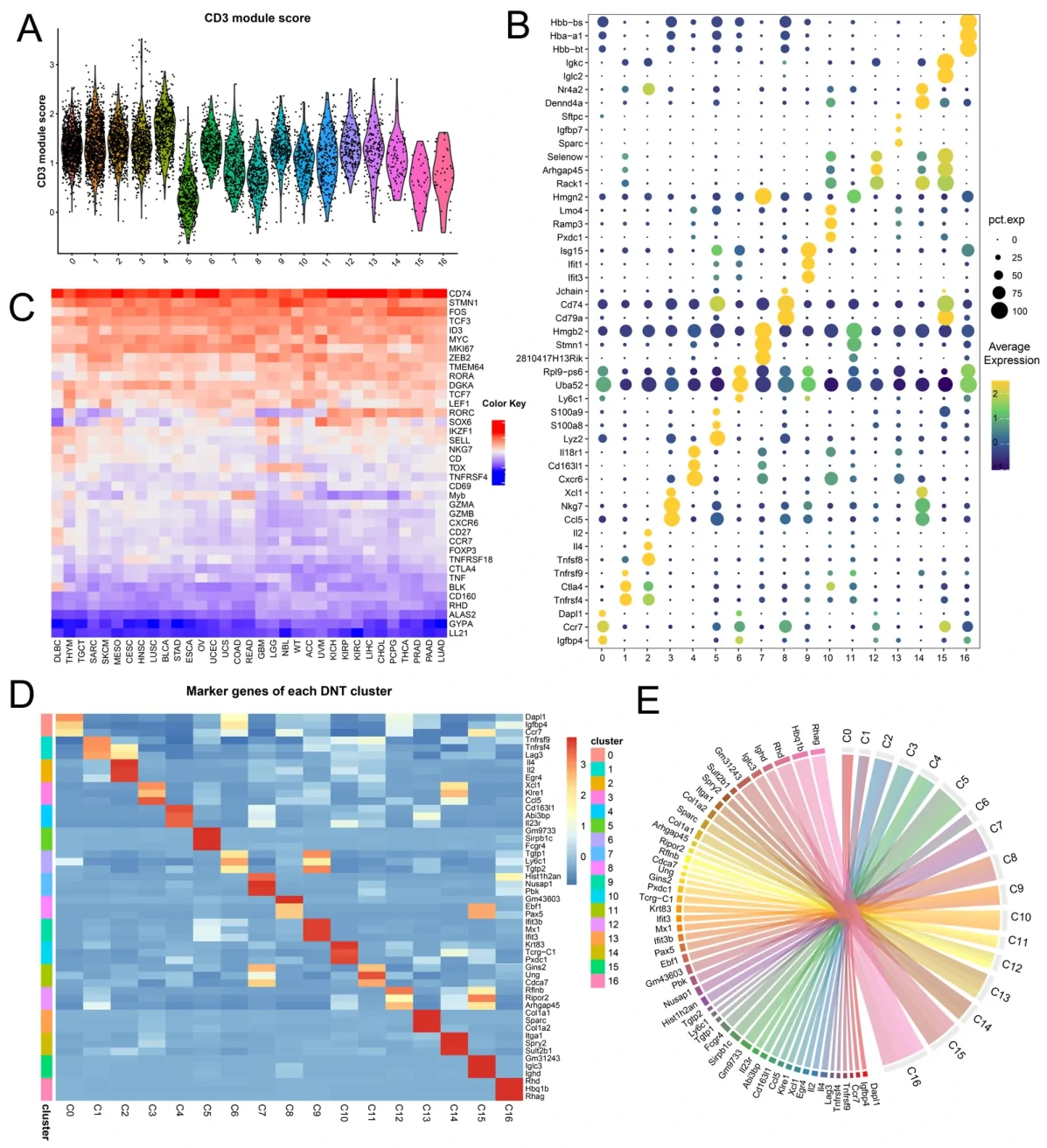
Marker gene characterization of mouse RNA defined DNT cell subsets. (**A**) Distribution of CD3 module scores across DNT cell clusters, calculated from Cd3d, Cd3e, and Cd3g. (**B**) Dot plot of representative marker gene expression across DNT cell clusters. Dot size indicates the percentage of cells expressing each gene, and color indicates average expression. (**C**) TCGA pan cancer expression profiles of human homologs of selected mouse DNT associated genes across cancer types. (**D**) Heatmap showing representative marker genes across DNT cell clusters. (**E**) Chord diagram showing the distribution of representative marker genes among DNT cell clusters.

Cluster specific marker gene analysis revealed substantial transcriptional differences among the identified mouse DNT cell clusters and supported annotation of their dominant transcriptional programs based on representative marker gene expression. The complete list of cluster specific marker genes identified across all DNT cell clusters is provided in Supplementary Table S2. The identified clusters were mainly characterized by naive, proinflammatory, cytotoxic, proliferative, and myeloid associated transcriptional programs. Clusters 0, 2, and 6 exhibited prominent naive related expression patterns, whereas clusters 1, 4, 10, 13, and 14 were associated with proinflammatory signatures. Cytotoxic transcriptional programs were primarily detected in clusters 3, 9, and 14. The simultaneous enrichment of proinflammatory and cytotoxic genes in cluster 14 indicated that this cluster represented a transcriptional state containing features of both inflammatory activation and cytotoxic effector activity. Clusters 7 and 11 were characterized by proliferative features, while cluster 5 displayed a prominent myeloid associated and innate inflammatory transcriptional program. This cluster showed coordinated enrichment of Lyz2, S100a8, S100a9, Tyrobp, Fcer1g, and Lst1, indicating a transcriptionally unusual population with concurrent T cell associated and myeloid associated signals. Because cluster 5 retained T cell associated transcriptional features, this population was retained as an RNA defined DNT candidate state with myeloid associated features, while residual doublets or myeloid contamination could not be completely excluded [12]. A CD3 module score based on Cd3d, Cd3e, and Cd3g was calculated for each cell. The CD3 module score was detected across all identified clusters and was used to summarize CD3 family transcriptional signals across the retained population, as shown in Figure 3A. The individual expression patterns of Cd3d, Cd3e, and Cd3g are shown in Supplementary Figure S1A–C. Considered together with Trac and Trbc2 expression and the absence of detectable Cd4, Cd8a, and Cd8b1 transcripts, these patterns were consistent with the intended T cell transcriptional profile of the RNA defined DNT population. To further evaluate potential unconventional T cell associated transcriptional signals, γδ T cell and MAIT associated TCR genes were examined in the sample level candidate DNT matrices before integration. As expected from the initial selection criteria, Trdc and Klrb1c were not detected among the retained cells. Among the additional evaluable genes, Trgj1, Trgj2, Trgj3, Trgj4, and Traj33 were also undetectable, whereas Trgv2 and Trav1 were detected at low frequencies of 2.784% and 0.524%, respectively. Trav1 expression was not accompanied by detectable Traj33 expression, providing no evidence of the canonical Trav1-Traj33 MAIT associated TCR combination. In the final processed DNT object, residual Trgv2 expression was restricted to a small subset of cells. A post hoc sensitivity assessment excluding Trgv2 positive cells showed that the major proinflammatory and cytotoxic marker patterns remained detectable within the existing cluster framework, including those characteristic of clusters 10 and 14, as shown in Supplementary Figure S2A and Supplementary Table S3. These results indicate that the principal marker defined DNT transcriptional programs were not primarily driven by the residual Trgv2 associated signal. This analysis was used to assess the contribution of residual unconventional T cell associated signals, because the absence of individual TCR associated transcripts may also reflect limited transcript detection in sparse scRNAseq data.

Visualization of representative marker genes showed that each DNT cell subset possessed a distinct expression profile, consistent with the transcriptional state annotations described above. As shown in Figure 3B, naive cluster 0 exhibited high expression of Igfbp4, Ccr7, and Dapl1, whereas proinflammatory cluster 4 was characterized by elevated expression of Cxcr6, Cd163l1, and Il18r1. Cytotoxic associated genes, including Ccl5, Nkg7, Xcl1, Gzmb, Cd7, and Cd160, were preferentially expressed in cytotoxic DNT subsets. Among these genes, Gzmb is a well established effector molecule associated with cytotoxic lymphocyte activity [13]. Ccl5 showed strong enrichment in cytotoxic cluster 3, whereas Cxcr6 was prominently enriched in proinflammatory cluster 4. Together with their differential expression magnitude, expression prevalence, and cluster specificity, these patterns supported the subsequent selection of Ccl5 and Cxcr6 as additional virtual knockout targets representing the cytotoxic and proinflammatory states, respectively. The coordinated expression of these effector related genes further distinguished cytotoxic DNT populations from naive and proinflammatory subsets and indicated marked transcriptional diversity among the identified clusters. To explore the potential relevance of mouse DNT associated marker genes in human cancer, the corresponding human homologs were examined in bulk transcriptomic datasets from 33 TCGA cancer types. Pan cancer analysis revealed that CD74, STMN1, FOS, TCF3, and ID3 were broadly upregulated across multiple cancer types, whereas GZMB, CXCR6, CD27, CCR7, and FOXP3 generally showed lower expression levels, as illustrated in Figure 3C. These results showed that human homologs corresponding to selected mouse DNT associated genes displayed distinct expression patterns across cancer types, although the direction and magnitude of these changes varied among tumors. Because TCGA data were derived from bulk tumor tissues, this comparison provided a human cancer context for these genes. Their cellular origin in human tumors could not be determined from the TCGA data. The marker heatmap in Figure 3D showed that Il4 expression was markedly elevated in cluster 2 compared with the other DNT cell subsets. Figure 3E further summarized the distribution of representative marker genes across DNT cell clusters. Together with the naive related transcriptional features observed in this cluster, the selective expression of Il4 further distinguished cluster 2 from the remaining naive DNT populations and suggested that naive DNT subsets may differ in their cytokine expression profiles. Collectively, these results define the major transcriptional characteristics of mouse RNA defined DNT cell subsets and identify distinct marker gene combinations associated with naive, inflammatory, cytotoxic, proliferative, and myeloid associated states.

### 2.4. Transcriptional Relationships Among DNT Cell Subsets

**Figure 4:**
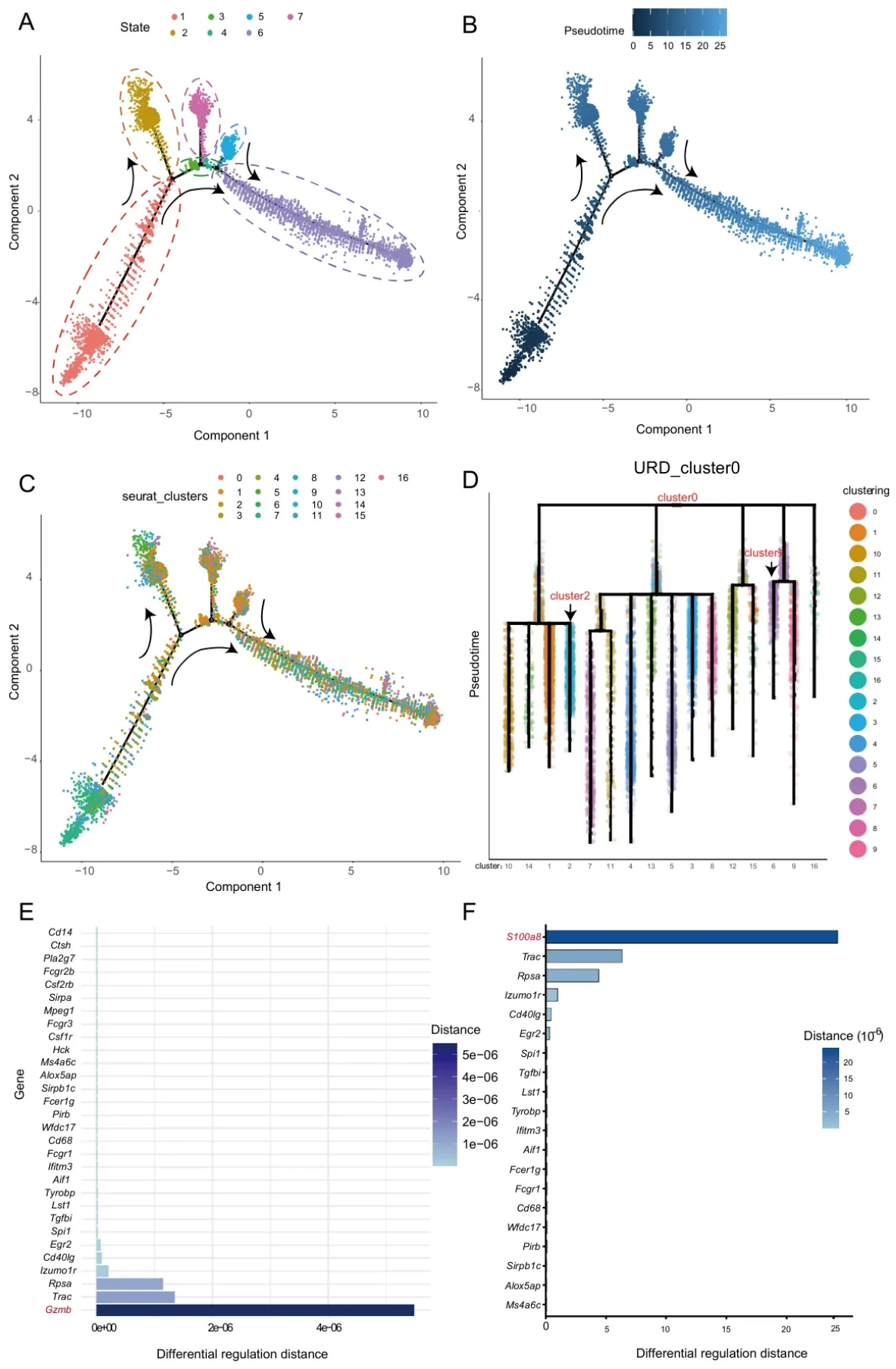
Trajectory inference and virtual knockout analysis of mouse RNA defined DNT cell subsets. (**A**) Monocle inferred trajectory of DNT cells colored by trajectory state. (**B**) Pseudotime ordering of DNT cells along the reconstructed trajectory. (**C**) Distribution of DNT clusters across the inferred trajectory. (**D**) URD based hierarchical trajectory reconstruction using cluster 0 as the root population. (**E**) Virtual knockout of Gzmb showing genes with the largest differential regulation distances. (**F**) Virtual knockout of S100a8 showing genes with the largest differential regulation distances.

To further characterize the transcriptional relationships among transcriptionally distinct mouse DNT cell subsets, pseudotime trajectory inference was performed using Monocle. The reconstructed trajectory displayed a continuous and branched transcriptional structure with partial overlap among clusters, as shown in Figure 4A–C. DNT cells were distributed across several major trajectory states, with three prominent bifurcation points identified along the inferred path. Cells from different clusters partially overlapped within the central regions of the trajectory but gradually separated toward distinct branches. These patterns indicated both shared and divergent transcriptional relationships among the identified DNT cell subsets. The pseudotime gradient represented the relative transcriptional ordering of cells along the reconstructed trajectory. To further relate the inferred trajectory to the transcriptional state annotation of DNT cells, the annotated cell states were mapped onto the same trajectory. Naive, inflammatory, cytotoxic, proliferative, and myeloid associated DNT states showed distinct but partially overlapping distributions along the trajectory, as shown in Supplementary Figure S1D. However, the trajectory represents an inferred transcriptional ordering of cells and does not directly establish a chronological differentiation process or lineage relationship [14]. To further examine the hierarchical organization of DNT cell states, URD based trajectory reconstruction was performed, with cluster 0 selected as the root population because of its prominent naive transcriptional features. The resulting trajectory also displayed a branched organization and identified four major transcriptional routes within the DNT cell population, as shown in Figure 4D. Clusters 2 and 6 were positioned on relatively independent branches, distinguishing these subsets from clusters distributed along the remaining shared trajectories. Their separate locations indicated that clusters 2 and 6 possessed transcriptional programs that differed from those represented along the other major trajectory branches.

In contrast, the other branches contained multiple clusters arranged along continuous routes, with adjacent subsets showing closer transcriptional relationships and more distant clusters occupying separate terminal regions. The overall topology obtained from URD was therefore consistent with the branched structure observed in the Monocle analysis. Together, the two trajectory analyses revealed a branched transcriptional organization in which different DNT cell subsets showed both shared and divergent state relationships. These inferred trajectories provide a framework for describing the relative transcriptional relationships among naive, inflammatory, cytotoxic, proliferative, and myeloid associated DNT states.

### 2.5. Virtual Knockout Analysis of Representative DNT Associated Genes

To examine the predicted regulatory consequences of perturbing representative DNT associated genes, an in silico virtual knockout analysis was performed. Among genes showing cluster associated differential expression across DNT subsets, Gzmb and S100a8 were initially selected as representative perturbation targets. Gzmb showed preferential expression in cytotoxic DNT subsets and was selected to represent the cytotoxic transcriptional program [15]. S100a8 was prominently enriched in cluster 5 and was selected to represent the myeloid associated and innate inflammatory transcriptional program of this cluster [16]. The initial selection considered both cluster associated expression patterns and the established biological functions of these genes.

Gzmb was additionally supported by the established role of granzyme B in cytotoxic lymphocyte mediated target cell killing [17]. Granzyme B contributes to target cell killing through proteolytic activation of cell death pathways and has also been reported to act on intracellular and extracellular substrates and participate in broader immunoregulatory processes [18]. To provide an additional quantitative basis for target selection, cluster specific marker genes were further evaluated according to differential expression significance, expression magnitude, expression prevalence, and cluster specificity, as described in the Materials and Methods. Based on these criteria, Ccl5 and Cxcr6 were selected as additional targets representing the cytotoxic and proinflammatory transcriptional states, respectively. Virtual knockout of Gzmb showed that Gzmb itself had the largest differential regulation distance, followed by Trac, Rpsa, Izumo1r, and several other genes. In the scTenifoldKnk framework, virtual removal of Gzmb modifies the inferred gene regulatory network, and differential regulation is evaluated from changes in gene positions between the original and perturbed network manifolds. Therefore, these changes indicate altered inferred network relationships after Gzmb perturbation [19], as shown in Figure 4E. To further examine the biological processes associated with Gzmb perturbation, an additional analysis was performed within the cytotoxic DNT population, including clusters 3, 9, and 14. Genes were ranked according to their standardized differential regulation scores after virtual Gzmb knockout and analyzed using Gene Ontology Biological Process gene set enrichment analysis. Significant enrichment was observed for interferon mediated signaling, type I interferon mediated signaling, response to interferon beta, antiviral responses, regulation of cytokine mediated signaling, and T cell differentiation related processes, as shown in Supplementary Figure S2B. Additional significant processes included alpha beta T cell activation, regulation of T cell activation, and positive regulation of immune effector processes. The complete significant GO Biological Process enrichment results are provided in Supplementary Table S4. These results indicate that virtual Gzmb perturbation in cytotoxic DNT cells was associated with broader network changes involving interferon related responses, cytokine signaling, and T cell associated immune programs. These enriched processes summarize the functional characteristics of genes showing stronger network perturbation. Consistent with the established involvement of S100a8 in inflammatory and myeloid associated processes [20], virtual knockout of S100a8 showed the largest differential regulation distance for S100a8 itself, followed by Trac, Rpsa, Izumo1r, and additional genes, as shown in Figure 4F. The additional Ccl5 analysis identified Ccl5, Trac, Rpsa, Izumo1r, and other genes among those showing prominent regulatory changes, whereas virtual knockout of Cxcr6 identified Cxcr6, Rpsa, Trac, and additional genes among those with the largest differential regulation distances, as shown in Supplementary Figure S1E,F. Although the magnitude and ranking of affected genes differed among the four targets, several genes, particularly Trac and Rpsa, were repeatedly identified across the perturbation analyses. Together, these results indicate that virtual perturbation of representative state associated genes was accompanied by broader predicted changes in the inferred DNT gene regulatory network. The virtual knockout results were therefore interpreted as computational predictions of network perturbation. The association of the cytotoxic transcriptional states with Gzmb and Ccl5 is consistent with previous studies describing antitumor activity of DNT cells [21]. Related studies have also reported DNT-mediated cytotoxicity in hematological malignancies and engineered DNT based tumor targeting [22].

### 2.6. Ligand Receptor Based Cell Communication Analysis of DNT Cell Subsets

**Figure 5:**
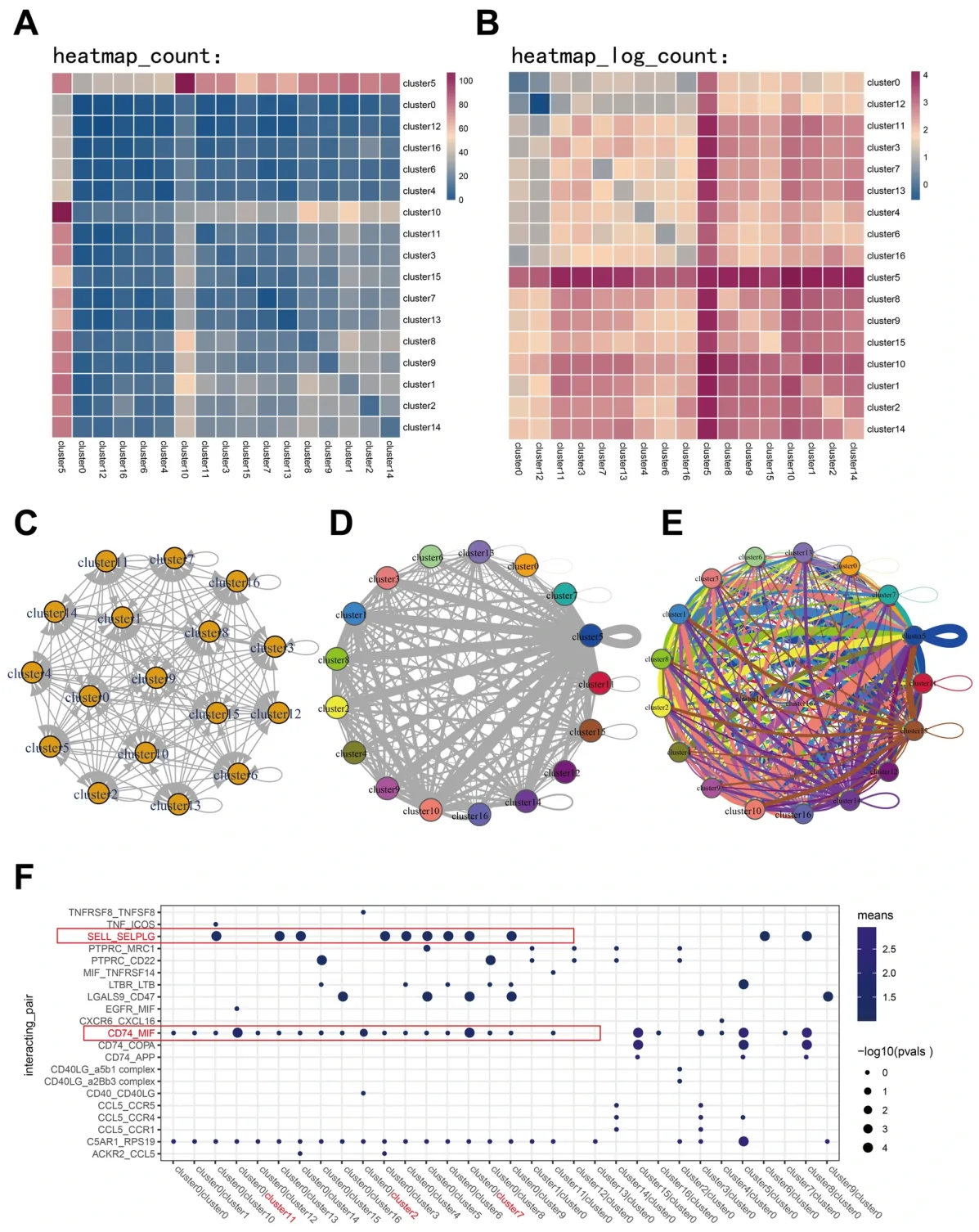
Cell communication analysis among mouse RNA defined DNT cell subsets. (**A**) Heatmap showing the number of predicted ligand receptor interactions among DNT clusters. (**B**) Log transformed heatmap showing the number of predicted ligand receptor interactions among DNT clusters. (**C**) Network visualization of global inter cluster communication. (**D**) Chord diagram showing major predicted communication relationships among selected DNT subsets. (**E**) Network visualization showing predicted communication relationships among DNT clusters. (**F**) Bubble plot showing representative ligand receptor interactions, including SELL SELPLG and CD74 MIF interactions.

Ligand receptor based communication analysis revealed an extensive but uneven interaction network among the transcriptionally distinct DNT cell subsets. The global interaction profile revealed that DNT subsets were connected through a complex communication network, suggesting that these cells may participate in distinct patterns of intercellular signaling. The interaction count heatmap showed clear heterogeneity among clusters, as shown in Figure 5A. Cluster 5 exhibited the highest number of predicted ligand receptor interactions and showed extensive connections with multiple DNT cell subsets. This cluster therefore represented the most highly connected population within the overall communication network. Together with its innate inflammatory and myeloid associated transcriptional features, the high predicted connectivity of cluster 5 indicates a distinct communication profile within the analyzed DNT compartment. However, these potential functions remain predictions derived from transcriptomic and ligand receptor analyses and should therefore be interpreted cautiously. In contrast, cluster 10 displayed a relatively small number of predicted interactions and showed a more restricted communication profile. The log transformed interaction heatmap further confirmed the unequal distribution of predicted interaction numbers across the DNT subsets, as shown in Figure 5B. Several clusters exhibited strong and broadly distributed interactions with multiple partner populations, whereas others showed weaker or more selective interaction patterns. These results demonstrated that the identified DNT subsets differed not only in their transcriptional characteristics but also in the number and strength of their predicted intercellular interactions. Network visualization provided a direct representation of these relationships. The global communication network contained dense connections among multiple DNT clusters, but the distribution of these connections was not uniform, as shown in Figure 5C–E. Clusters with large numbers of links occupied central positions in the network, whereas clusters with fewer interactions were located in less connected regions. Cluster 5 showed a particularly prominent network position, consistent with the interaction count analysis, while cluster 10 remained comparatively weakly connected. Analysis of individual ligand receptor pairs further identified both broadly distributed and cluster selective communication patterns within the DNT cell compartment. In the communication network associated with naive cluster 0, the SELL and SELPLG interaction was detected across multiple interacting clusters, as shown in Figure 5F [23]. The widespread distribution of this interaction distinguished SELL and SELPLG as one of the commonly detected signaling pairs among the analyzed DNT subsets. In contrast, the Cd74 and MIF interaction showed a more restricted distribution and was predominantly detected in clusters 7 and 11 [24]. Both clusters had previously been characterized by proliferative transcriptional features. The preferential detection of the Cd74 and MIF pair in these clusters therefore revealed an association between this predicted communication pattern and proliferative DNT cell states. Other ligand receptor interactions also varied in their distribution and predicted strength among the clusters, further contributing to the heterogeneous structure of the communication network.

Taken together, these results show that mouse RNA defined DNT cell subsets possess distinct predicted communication profiles. Some ligand receptor pairs were shared across several clusters, whereas others were concentrated within specific transcriptional subsets. The unequal interaction counts, variable interaction strengths, and cluster selective signaling patterns provide additional evidence that the mouse DNT compartment contains transcriptionally heterogeneous populations with distinct predicted communication profiles.

## 3. Discussion

This study established an integrated single cell data fusion strategy for the systematic reconstruction and characterization of rare DNT cell populations across multiple tissues. Source level quality assessment and RNA based DNT screening were performed before integration so that the retained RNA defined DNT population could be evaluated before subsequent clustering and functional analyses. By applying stringent marker based extraction criteria and integrating single cell RNA sequencing datasets from multiple sources, this framework increased the number of analyzable DNT cells. At the same time, the integration procedure was designed to facilitate joint analysis across tissue sources, address heterogeneity among the integrated datasets, and address the challenges of limited cell numbers and substantial inter dataset heterogeneity in rare immune cell research. The integrated analytical workflow encompassed batch correction, unsupervised clustering, marker based annotation, pan cancer comparative analysis, trajectory inference, virtual gene knockout, and ligand receptor based cell communication analysis, providing a multidimensional characterization of DNT cell diversity. Through this strategy, multiple transcriptionally distinct DNT cell clusters were identified, encompassing naive, proinflammatory, cytotoxic, proliferative, and myeloid associated transcriptional states [25]. Among these states, cluster 5 was characterized by coordinated enrichment of Lyz2, S100a8, S100a9, Tyrobp, Fcer1g, and Lst1, indicating a prominent innate inflammatory and myeloid associated transcriptional program. Because this population retained T cell associated transcriptional features, it was retained as an RNA defined DNT candidate state with myeloid associated features, although residual doublets or myeloid contamination cannot be completely excluded. Consistent with this interpretation, virtual knockout of S100a8 produced a distinct regulatory perturbation pattern within the inferred DNT gene regulatory network, while cluster 5 also exhibited extensive predicted ligand receptor connectivity. These complementary features suggest that cluster 5 represents a transcriptionally unusual candidate population with mixed T cell and myeloid associated signals and predicted intercellular communication activity. In addition, Ccl5 and Cxcr6 were included as additional virtual knockout targets selected using predefined quantitative marker criteria, representing the cytotoxic and proinflammatory states, respectively. Their perturbation profiles provided complementary regulatory information for these DNT transcriptional programs. Together, these findings further illustrate the transcriptional and predicted regulatory diversity present within the integrated DNT landscape. The tissue composition analysis further indicated that the identified RNA defined DNT states differed in their representation across tissue sources. Although several transcriptional states were broadly represented across tissues, others were more concentrated in a limited number of tissues. These findings describe differences in the distribution of integrated DNT states across tissue sources but do not establish that tissue origin directly drives the underlying transcriptional programs. Differences in source datasets and experimental context should also be considered when interpreting these patterns.

These transcriptionally distinct subsets collectively delineated the molecular heterogeneity of DNT cells across the integrated datasets and provided a foundation for further investigation of their potential state relationships and intercellular signaling patterns [11]. Trajectory analysis suggested an inferred transcriptional continuum linking naive associated states with inflammatory and cytotoxic effector programs, whereas cell communication analysis identified heterogeneous predicted signaling networks among different DNT subsets. Importantly, this inferred ordering primarily reflects transcriptional similarity and state relationships [26]. Collectively, these findings highlight the complexity of DNT cell populations across tissues and support the utility of integrated single cell analysis for resolving rare immune cell populations in complex biological and disease contexts [27]. Because the integrated single cell analyses were based on mouse datasets, the identified transcriptional states primarily characterize mouse RNA defined DNT cells. The TCGA analysis of corresponding human homologs provides a complementary human cancer related context, while potential species differences in tissue environment and transcriptional regulation should be considered when relating these mouse defined states to human DNT biology. Several considerations should be taken into account when interpreting the present findings. The integrated analysis was based on publicly available single cell RNA sequencing datasets derived from multiple tissues and experimental settings. Previous single cell studies provide methodological precedent for transcriptome based identification of DNT populations, but this approach does not eliminate the uncertainty associated with transcript dropout. In these studies, DNT cells were identified within an established T cell population before evaluation of CD4 and CD8 transcript status and additional lineage associated signals. The present study followed the same general analytical sequence. T cell identity was first established using multiple T cell associated transcripts, followed by evaluation of coreceptor and lineage associated transcripts. The retained population was further assessed using the CD3 module score and unconventional T cell associated transcriptional signals. Although the integration strategy facilitated joint analysis across tissue sources, differences in sample source and experimental context should be considered when interpreting cross tissue patterns. Because unfiltered droplet level count matrices were not uniformly available across the public datasets, ambient RNA correction could not be applied consistently, and the potential contribution of residual background transcripts cannot be completely excluded [28]. The retained population was also evaluated for unconventional T cell associated transcriptional signals. The major marker defined transcriptional patterns remained detectable after removal of Trgv2 positive cells, indicating that these patterns were not primarily driven by the detectable Trgv2 positive population. Nevertheless, transcript dropout remains an inherent limitation of droplet based single cell RNA sequencing. The absence of individual lineage associated transcripts cannot definitively exclude γδ T, MAIT, NKT, or other unconventional T cell populations. Recently activated conventional CD4 or CD8 T cells with reduced or undetected coreceptor transcripts also cannot be completely excluded. Therefore, the analyzed population is referred to as RNA defined DNT cells and should not be considered equivalent to DNT cells confirmed by protein level immunophenotyping or paired TCR profiling. Prospective immunophenotyping and paired TCR analysis will be required for more definitive validation of cell identity. Future studies using appropriately matched reference based annotation could compare the resulting cell assignments with those obtained using marker based strategies, including the present integrated analysis [29]. This comparison may provide an additional assessment of the robustness of DNT cell identification across different annotation strategies. Furthermore, the inferred transcriptional states and trajectory relationships primarily reflect transcriptional similarities and ordering among DNT cells. Thus, these analyses are most appropriately interpreted as a framework for describing potential state relationships and regulatory features within the DNT landscape. Virtual Gzmb perturbation provided additional insight into the regulatory features associated with the cytotoxic DNT state. Genes showing stronger network perturbation after virtual Gzmb knockout were enriched in interferon related responses, antiviral immune processes, cytokine signaling, and T cell differentiation and activation programs. These findings suggest that the cytotoxic DNT transcriptional state represented by Gzmb is associated with broader immune regulatory programs involving interferon responses, antiviral immunity, cytokine signaling, and T cell related functions. The scTenifoldKnk results were therefore interpreted at the level of inferred network relationships. Further experimental studies will be required to determine the biological relationships underlying these predicted network changes. Future studies could extend this computational framework through the analysis of independent single cell datasets, spatial transcriptomic datasets [30], disease specific cohorts, and additional cross species datasets to further evaluate the reproducibility and biological relevance of the identified DNT cell states [31]. Comparison with human DNT single cell datasets will be particularly useful for determining which transcriptional states and functional programs are shared between mice and humans. Graph based computational models may also facilitate the integration of DNT cell transcriptional signatures with additional regulatory information and improve the prioritization of disease-associated regulators [32]. Such extensions will help clarify the roles of DNT cells in tissue immune homeostasis, inflammation [33], tumor immunity, and disease progression, and may provide new insights into the identification of DNT cell related biomarkers and candidate molecular regulators associated with disease [34].

## 4. Materials and Methods

### 4.1. Data Collection and RNA Based Identification of Mouse DNT Cells

Large scale, multi source mouse single cell RNA sequencing datasets were obtained from publicly available Gene Expression Omnibus resources [35]. Publicly available mouse single cell transcriptomic datasets were searched in GEO using Mus musculus together with the keywords “scRNAseq” OR “single-cell RNA-seq” OR “scRNA-seq” OR “single-cell RNA-sequencing” OR “single cell RNA sequencing”. The sample descriptions provided in GEO and the corresponding source studies were then examined individually to assess their suitability for the present analysis. Based on this evaluation, a total of 66 publicly available mouse GEO samples were included in this study, and their corresponding accession numbers and sample information are summarized in Supplementary Table S1. The datasets were first reviewed according to tissue origin, sample information, sequencing platform, availability of gene expression matrices, and cell level metadata. Datasets with accessible expression matrices and sufficient sample and tissue annotation were retained for analysis. Datasets or samples with insufficient metadata or unclear tissue information were excluded during the initial data curation. Gene symbols were then standardized across datasets to ensure consistency before cell selection. For comparison with human pan cancer transcriptomic data, mouse genes were converted to their corresponding human homologs using matched gene annotations and established bioinformatics resources [36].

Candidate DNT cells were identified separately within each source dataset. A uniform RNA based marker gene strategy was applied to all datasets [8]. In the mouse single cell study by Islam et al., DN cells were identified within an established T cell population according to CD4, CD8a, and CD8b1 transcript expression. CD3 expression and transcriptomic feature detection were also examined to assess the retained DN population, and Trgv2 expression was evaluated to identify potential γδ T cells. In another large scale single cell study, Hao et al. first established the T cell compartment and subsequently extracted DNT cells for downstream analysis. These studies used the same general sequence of establishing T cell identity before evaluating coreceptor status and additional lineage associated signals. Following this strategy, cells in the present study were first required to show detectable expression of the T cell associated genes Ptprc, Cd3d, Cd3e, Cd3g, Trac, and Trbc2. Candidate cells were then required to show no detectable expression of Cd4, Cd8a, Cd8b1, Trdc, or Klrb1c. Thus, DNT cell selection combined multiple positive and negative transcriptional criteria. The retained cells were further evaluated using the CD3 module score, Trac and Trbc2 expression, and unconventional T cell associated transcripts. Trgv2 positive cells were additionally removed in a sensitivity assessment. These criteria provided an operational definition for enriching RNA defined DNT cells from retrospective scRNA sequencing datasets. Because the available feature spaces differed among source datasets, genes not represented in an individual matrix were classified as not evaluable. The final processed DNT object was also examined for residual unconventional T cell associated transcriptional signals. A post hoc sensitivity assessment was subsequently performed after exclusion of cells with detectable Trgv2 expression while retaining the existing cluster assignments. These analyses served as post selection assessments of residual unconventional T cell associated transcriptional signals. In particular, the absence of an individual TCR associated transcript was not considered sufficient by itself to establish lineage negativity, because sparse transcript detection may limit the evaluation of individual genes in droplet based scRNAseq data. After marker based selection, the corresponding dataset, sample, and tissue information were retained for each candidate DNT cell. Candidate cells passing the predefined criteria were then combined across source datasets into a unified gene expression matrix for subsequent single cell analysis.

### 4.2. Quality Control, Normalization, Integration, and Clustering

Each source dataset was first processed separately and evaluated for cell quality before DNT cell screening [37]. Because the public datasets originated from different studies and differed in cell number, sequencing depth, and expression complexity, quality assessment was performed separately for each source dataset. During initial data processing, genes were retained only when detected in at least three cells, and cells were required to contain at least 200 detected features. These criteria were used to reduce the contribution of extremely sparse genes and cells containing very limited transcriptomic information. Cell quality was evaluated using the number of detected genes, total transcript counts, and the proportion of mitochondrial transcripts [38]. Mitochondrial genes were identified using the pattern “^mt-”, and the PercentageFeatureSet function was used to calculate the proportion of total cellular counts contributed by these genes. The distributions of these quality control features were examined separately within each source dataset to characterize cell quality in terms of transcriptomic complexity, feature counts, and mitochondrial transcript levels. These source specific quality assessments were completed before subsequent DNT cell screening [39]. Dataset specific quality control thresholds were determined before DNT cell screening according to the distributions of detected features, total transcript counts, and mitochondrial transcript proportions. The thresholds applied to individual datasets are provided in Supplementary Table S1. Candidate DNT cells passing the predefined RNA based marker criteria were subsequently combined across datasets, and the same quality related features were reassessed in the pooled candidate DNT population before normalization and integration. Only cells retained after source-level quality assessment and RNA based DNT screening were included in the subsequent integration procedure. Following quality control, gene expression values were normalized using the NormalizeData function in Seurat with the LogNormalize method and a scale factor of 10,000 [37]. For each cell, gene counts were normalized to the total cellular counts, multiplied by 10,000, and log-transformed. Highly variable genes were identified using the FindVariableFeatures function with the vst method, and the top 2000 variable genes were retained. Expression values were then centered and scaled using the ScaleData function, with all genes in the Seurat object included in the scaling step. Principal component analysis was performed using the highly variable genes, and 50 principal components were computed.

Data integration was subsequently performed using Harmony on the principal component representation, with tissue origin specified as the grouping variable in the RunHarmony function. This integration was used to facilitate joint analysis across tissue sources within a common low dimensional representation. Tissue labels were retained for subsequent descriptive analyses of cluster composition and tissue distribution. The corrected Harmony dimensions were used instead of the uncorrected principal components for subsequent neighborhood construction, clustering, and visualization [40]. To examine the low dimensional distributions before and after Harmony integration, the uncorrected PCA representation and the Harmony corrected representation were visualized according to tissue origin. The first 10 Harmony dimensions were used for downstream analyses. A shared nearest neighbor graph was constructed using the selected Harmony dimensions, and graph based clustering was performed with the FindClusters function in Seurat using a resolution of 0.7. UMAP was generated from the first 10 Harmony dimensions to visualize the transcriptional distribution of DNT cells [41]. The original dataset, sample, and tissue information was retained throughout the analysis and was used to examine the tissue composition and distribution of the identified DNT cell clusters. To compare cluster composition across tissues, the number of RNA defined DNT cells assigned to each cluster was divided by the total number of RNA defined DNT cells from the corresponding tissue. The resulting within tissue normalized proportions were visualized as a tissue by cluster heatmap. To further evaluate the tissue distribution of individual clusters, the proportion of cells contributed by each tissue was calculated relative to the total number of cells in that cluster. The Shannon entropy was then calculated from these tissue proportions and normalized by the maximum entropy expected for eight tissues. The tissue specificity score was defined as one minus the normalized Shannon entropy, with higher values indicating a more restricted tissue distribution and lower values indicating a broader distribution across tissues.

### 4.3. Marker Gene Identification and Transcriptional State Annotation

Cluster specific marker genes were identified using the FindAllMarkers function in Seurat. Each cluster was compared with all remaining clusters using the Wilcoxon rank sum test [42]. Marker genes were interpreted according to their differential expression patterns, expression prevalence, and cluster specificity, with reference to established approaches for single cell differential expression and marker identification [43]. Transcriptional state annotation of DNT cell clusters was performed by integrating cluster specific marker genes, canonical immune cell markers, and published reference signatures. To further evaluate the T cell associated transcriptional characteristics of the retained DNT cells, a CD3 module score was calculated based on Cd3d, Cd3e, and Cd3g using the AddModuleScore function implemented in Seurat. For each cell, the module score represents the average normalized expression of the three CD3 genes relative to the average expression of matched control genes selected from genes with similar overall expression levels. The resulting CD3 module scores were compared across the identified DNT cell clusters. DNT subsets were annotated as naive, proinflammatory, cytotoxic, proliferative, or myeloid associated states according to their dominant transcriptional features. The annotations were based on coordinated expression patterns across multiple representative genes. The myeloid associated state was defined by coordinated enrichment of innate inflammatory and myeloid associated genes. Representative genes included Lyz2, S100a8, S100a9, Tyrobp, Fcer1g, and Lst1. This state was interpreted as an RNA defined DNT state showing a distinct innate inflammatory transcriptional program. Cytotoxic states were characterized by enrichment of cytotoxic effector associated genes, including Gzmb, Nkg7, and Cd160, whereas proinflammatory states were defined by coordinated expression of inflammation-associated markers identified in the cluster specific analysis. Gzmb was included among the representative cytotoxic markers based on its established role as an effector molecule of cytotoxic lymphocytes [17].

### 4.4. Pan Cancer Comparison of DNT Associated Marker Genes

To explore the potential relevance of mouse DNT subset associated marker genes in human cancer, transcriptomic profiles covering 33 human cancer types were obtained from TCGA [44]. TCGA pan cancer resources have been widely used to characterize molecular and immune related variation across multiple cancer types [44,45]. Mouse marker genes identified in the DNT cell analysis were converted to their corresponding human homologs before comparison. Expression levels of these human homologs were analyzed across cancer types. Differential expression patterns between tumor tissues and matched or available normal tissues were evaluated according to data availability. Genes showing broad upregulation or downregulation across multiple cancer types were interpreted as candidate tumor associated immune regulatory genes. The pan cancer analysis was therefore used to provide a human cancer related context for selected mouse DNT associated genes at the gene expression level, while their cellular origin in human tumors could not be determined from the bulk TCGA data.

### 4.5. Trajectory Inference of DNT Cell Subsets

Pseudotime trajectory analysis was performed to infer potential cell state relationships among DNT subsets [46]. Monocle was used to reconstruct an inferred transcriptional trajectory based on selected variable genes and cluster associated marker genes. Cells were ordered along pseudotime, and trajectory branches were visualized according to cell state, pseudotime value, and Seurat defined cluster identity. The transcriptional state annotations of DNT cell subsets were additionally mapped onto the Monocle trajectory to visualize the distribution of naive, inflammatory, cytotoxic, proliferative, and myeloid associated states. Clusters with naive associated transcriptional features were used to guide root state selection. The interpretation of branched pseudotime structures was also considered in relation to established single cell lineage inference frameworks [47]. To further evaluate hierarchical cell state relationships, URD based trajectory reconstruction was performed [48]. Cluster 0 was selected as the root population because it showed naive characteristics and enrichment of genes associated with naive or early immune states. The URD trajectory was used to infer branched routes from naive DNT states toward inflammatory, cytotoxic, proliferative, and myeloid associated programs. Monocle and URD results were interpreted together to identify shared transcriptional transition patterns.

### 4.6. Virtual Knockout Analysis

Virtual knockout analysis was performed using the scTenifoldKnk R package to estimate the predicted regulatory consequences of gene specific perturbation in DNT cells [19]. scTenifoldKnk reconstructs a single cell gene regulatory network from the input expression matrix and generates a corresponding perturbed network by simulating loss of a selected gene. Gzmb and S100a8 were initially selected as representative genes of the cytotoxic and myeloid associated inflammatory transcriptional programs, respectively, based on their cluster associated expression patterns and established biological relevance. Additional targets were subsequently selected using quantitative marker criteria, as described below. To further evaluate the influence of target selection on the virtual knockout analysis, an additional marker based selection procedure was applied. Candidate genes were screened according to statistical significance, differential expression magnitude, expression prevalence within the corresponding cluster, and expression specificity relative to the remaining cells. Candidate genes were required to have an adjusted *p*-value below 0.05, an average log2 fold change greater than 1, expression in more than 50 percent of cells within the corresponding cluster, and a difference between pct.1 and pct.2 greater than 0.30. Genes meeting these criteria were further considered according to their relevance to the major transcriptional states identified during cluster annotation. Ccl5 and Cxcr6 were used as additional perturbation targets representing the cytotoxic and proinflammatory transcriptional states, respectively.

For each target gene, the DNT cell gene expression matrix was used as input to construct the original single cell gene regulatory network according to the standard scTenifoldKnk workflow. A corresponding virtual knockout network was then generated by computationally perturbing the selected gene. The original and perturbed networks were compared to identify genes showing differential regulatory responses. Differential regulation was assessed according to the distance between the corresponding gene coordinates in the aligned original and perturbed network manifolds, with larger distances indicating greater predicted regulatory perturbation. The virtual knockout was therefore used to evaluate the network level response to computational removal of each selected gene. Differentially regulated genes identified by this procedure were interpreted as genes whose inferred network relationships changed after perturbation. Gzmb, S100a8, Ccl5, and Cxcr6 were analyzed separately using the same input processing procedure and parameter settings. Genes identified through network comparison were retained as perturbation associated genes for interpretation of the virtual knockout results. To further characterize the functional context of Gzmb associated network perturbation, an additional virtual knockout analysis was performed using cytotoxic DNT cells from clusters 3, 9, and 14. Genes were ranked according to the standardized differential regulation score generated by scTenifoldKnk after virtual Gzmb knockout. Gzmb itself was excluded from the ranked gene list before enrichment analysis. Gene Ontology Biological Process gene set enrichment analysis was performed using clusterProfiler and the mouse org.Mm.eg.db annotation database [49]. Enrichment significance was evaluated using Benjamini Hochberg adjusted *p*-values, with an adjusted *p*-value below 0.05 considered significant. Redundant Gene Ontology terms were reduced according to semantic similarity before visualization and interpretation. The significant nonredundant GO Biological Process terms after semantic similarity reduction are provided in Supplementary Table S4.

### 4.7. Ligand Receptor Based CellCommunication Analysis

Ligand receptor based intercellular communication analysis was performed using CellPhoneDB to infer potential signaling interactions among annotated DNT cell subsets [50]. The normalized gene expression matrix and the corresponding cell annotation file were used as input files. DNT cell clusters defined by Seurat clustering and functional annotation were used as cell group labels. Before analysis, mouse gene symbols were converted to their corresponding human orthologs and standardized to match the ligand receptor database used by CellPhoneDB. Genes with insufficient expression across the analyzed cell groups were filtered according to the default workflow. Interactions were retained when the ligand and receptor were expressed in a sufficient proportion of cells within the corresponding interacting groups. CellPhoneDB analysis was performed using the statistical analysis mode with default parameters, including the default expression proportion threshold and permutation based significance testing. The predicted ligand receptor interactions were evaluated according to interaction mean values and statistical significance. Interactions with *p*-values less than 0.05 were considered significant and were used for downstream visualization. The number of significant interactions and the relative communication strength were summarized among DNT cell subsets to assess global communication patterns. Heatmaps, network plots, chord diagrams, and bubble plots were generated to display overall interaction intensity, subset specific communication relationships, and representative ligand receptor pairs. This analysis was used to characterize potential intercellular signaling patterns among DNT subsets and to complement the clustering, marker annotation, trajectory inference, and virtual knockout analyses.

## Data Availability

The data presented inthis study are openly available in Gene Expression Omnibus (GEO) at [https://www.ncbi.nlm.nih.gov/geo/, accessed on 4 August 2026]. The relevant dataset information provided in Supplementary Materials. The analysis code is available from the corresponding author upon reasonable request.

## Supplementary Materials

The following supporting information can be downloaded at https://www.mdpi.com/article/10.3390/ijms27177966/s1.
