# Supplementary material for: Integrated Single Cell Analysis Reveals the Transcriptional Heterogeneity of Mouse Double Negative T Cells
Source: Int J Mol Sci. 2026 Sep 7;27(17):7966. doi: 10.3390/ijms27177966 (PMC13566193; doi:10.3390/ijms27177966)
Supplement: Supplementary file 1 [file ijms-27-07966-s001.zip › Supplementary Figure S1.pdf]

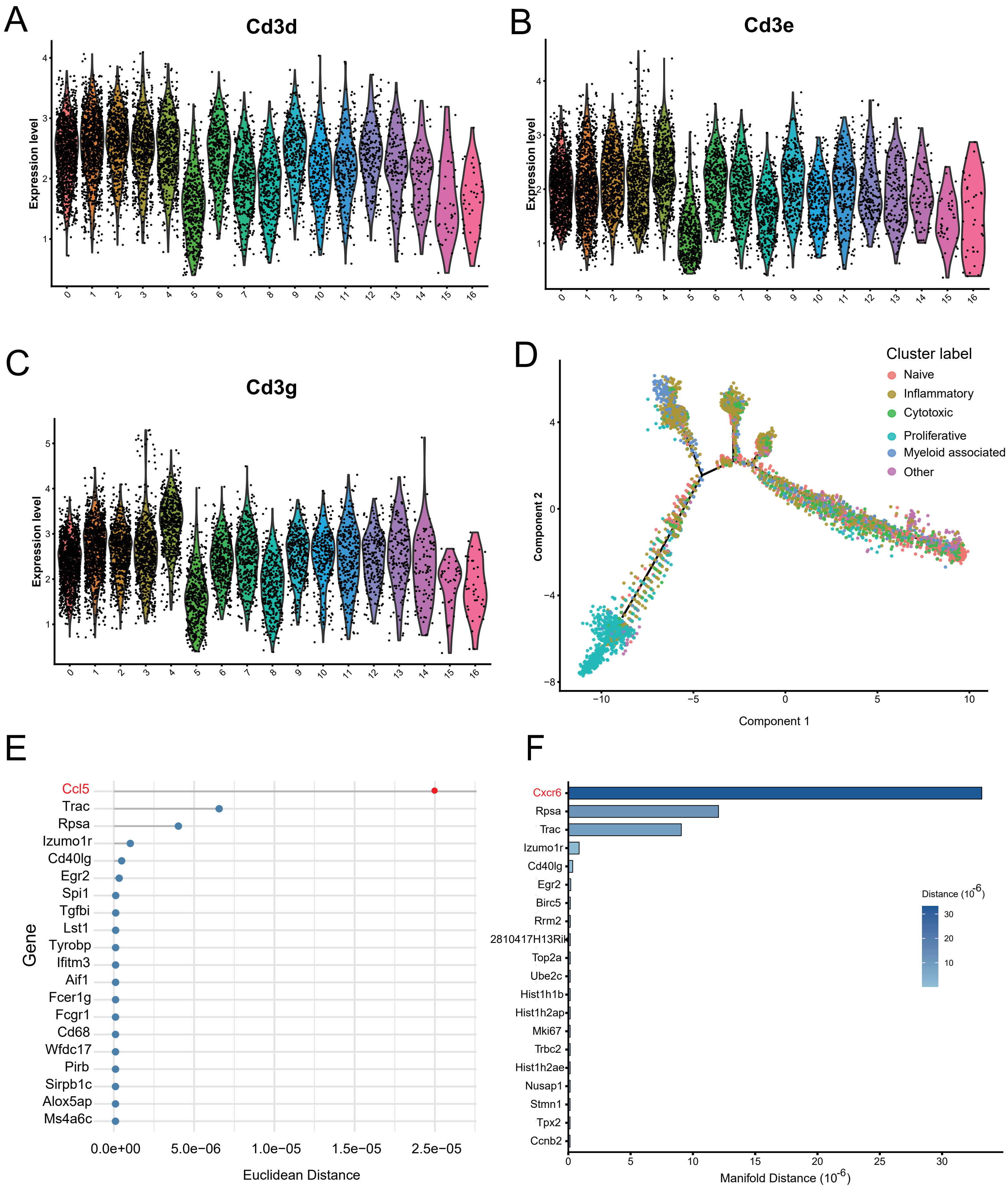
A
Cd3d
Expression level
0 1 2 3 4 5 6 7 8 9 10 11 12 13 14 15 16
B
Cd3e
Expression level
0 1 2 3 4 5 6 7 8 9 10 11 12 13 14 15 16
C
Cd3g
Expression level
0 1 2 3 4 5 6 7 8 9 10 11 12 13 14 15 16
D
Cluster label
Naive
Inflammatory
Cytotoxic
Proliferative
Myeloid associated
Other
Component 2
Component 1
E
Ccl5
Trac
Rpsa
Izumo1r
Cd40lg
Egr2
Spi1
Tgfbi
Lst1
Tyrobp
Ifitm3
Aif1
Fcer1g
Fcgr1
Cd68
Wfdc17
Pirb
Sirpb1c
Alox5ap
Ms4a6c
Gene
0.0e+00
5.0e-06
1.0e-05
1.5e-05
2.5e-05
Euclidean Distance
F
Cxcr6
Rpsa
Trac
Izumo1r
Cd40lg
Egr2
Birc5
Rrm2
2810417H13Rik
Top2a
Ube2c
Hist1h1b
Hist1h2ap
Mki67
Trbc2
Hist1h2ae
Nusap1
Stmn1
Tpx2
Ccnb2
Distance (10^-6)
Manifold Distance (10^-6)
